# Genome-wide characterization of phospholipase D family genes in allotetraploid peanut and its diploid progenitors revealed their crucial roles in growth and abiotic stress responses

**DOI:** 10.3389/fpls.2023.1102200

**Published:** 2023-01-20

**Authors:** He Zhang, Yang Yu, Shiyu Wang, Jiaxin Yang, Xin Ai, Nan Zhang, Xinhua Zhao, Xibo Liu, Chao Zhong, Haiqiu Yu

**Affiliations:** Peanut Research Institute, College of Agronomy, Shenyang Agricultural University, Shenyang, China

**Keywords:** PLDs, comparative genomics, molecular evolution, lipid metabolic network, growth and development, stress tolerance, *Arachis*

## Abstract

Abiotic stresses such as cold, drought and salinity are the key environmental factors that limit the yield and quality of oil crop peanut. Phospholipase Ds (PLDs) are crucial hydrolyzing enzymes involved in lipid mediated signaling and have valuable functions in plant growth, development and stress tolerance. Here, 22, 22 and 46 *PLD* genes were identified in *Arachis duranensis*, *Arachis ipaensis* and *Arachis hypogaea*, respectively, and divided into α, β, γ, δ, ε, ζ and φ isoforms. Phylogenetic relationships, structural domains and molecular evolution proved the conservation of *PLD*s between allotetraploid peanut and its diploid progenitors. Almost each *A. hypogaea PLD* except for *AhPLDα6B* had a corresponding homolog in *A. duranensis* and *A. ipaensis* genomes. The expansion of *Arachis PLD* gene families were mainly attributed to segmental and tandem duplications under strong purifying selection. Functionally, the most proteins interacting with AhPLDs were crucial components of lipid metabolic pathways, in which ahy-miR3510, ahy-miR3513-3p and ahy-miR3516 might be hub regulators. Furthermore, plenty of *cis*-regulatory elements involved in plant growth and development, hormones and stress responses were identified. The tissue-specific transcription profiling revealed the broad and unique expression patterns of *AhPLD*s in various developmental stages. The qRT-PCR analysis indicated that most *AhPLD*s could be induced by specific or multiple abiotic stresses. Especially, *AhPLDα3A*, *AhPLDα5A*, *AhPLDβ1A*, *AhPLDβ2A* and *AhPLDδ4A* were highly up-regulated under all three abiotic stresses, whereas *AhPLDα9A* was neither expressed in 22 peanut tissues nor induced by any abiotic stresses. This genome-wide study provides a systematic analysis of the *Arachis PLD* gene families and valuable information for further functional study of candidate *AhPLD*s in peanut growth and abiotic stress responses.

## Introduction

Cultivated peanut (*Arachis hypogaea* L.) is one of the most important grain legumes worldwide, ranking second in production among all grain legumes and fifth among oilseeds. China contributes the highest share and India ranks second by 36.48% and 13.97% in world production, respectively (United States Department of Agriculture (USDA) Foreign Agricultural Service, 2020). Peanut seeds, containing 40%-56% oil, 20%-30% protein and 10%-20% carbohydrates, have been primarily used to provide vegetable oil and proteins for human nutrition (Huang et al., 2015). In some Third World countries, peanut shows greater potential to reduce hunger and malnutrition as it is also a good source of quality fodder, calories, vitamins, minerals, and other antioxidant molecules (Krishna et al., 2015). However, majority of the world’s peanut is often grown on marginal soils with lesser inputs or intercropped with cereals in many developing countries. A huge gap is developed between its demand and supply due to the constrained quality and productivity resulting from various abiotic factors such as drought, salinity and temperature aberrations (Zhang et al., 2020; Shi et al., 2021). Thus, identification of key genes that can confer abiotic stress tolerance and can be utilized in biotechnological programs to generate improved varieties is an urgent requirement in peanut production (Raza et al., 2022a; Raza et al., 2022b).

One of the most crucial signaling networks for plants in response to multiple stimuli is mediated by lipid molecules (Rizwan et al., 2022a). Environmental cues can increase the activities of phospholipases and trigger the hydrolysis of membrane phospholipids, thus leading to the generation of different classes of lipids and lipid-derived signal messengers (Hou et al., 2016). Phospholipase D (PLD) represents a major family of membrane phospholipases in plants. It acts upon and cleaves the terminal phosphodiester bond of glycerophospholipids to produce phosphatidic acid (PA) and water-soluble free head group (Wang et al., 2012). *PLD* was first identified in plants as early as in 1940s (Hanahan and Chaikoff, 1947), but did not receive detailed attention until the 1980s (Bocckino et al., 1987). In the past two decades, the disclosure of numerous genomic resources facilitates the characterization of plant *PLD*s at a genome-wide level. The *PLD* gene families have been identified in *Arabidopsis* (*Arabidopsis thaliana*), rice (*Oryza sativa*), soybean (*Glycine max*), cotton (*Gossypium* spp.), rape (*Brassica napus* L.), chickpea (*Cicer arietinum*) and other plants successively (Qin and Wang, 2002; Li et al., 2007; Liu et al., 2010; Zhao et al., 2012; Tang et al., 2016a; Lu et al., 2019; Sagar et al., 2021). Commonly, all plant PLDs contain two conserved HKD domains (HxKxxxxD) responsible for hydrolysis activity and can be divided into three sub-classes based on the presence of different domains near the N-terminus: the PLDs with a calcium/phospholipid-binding C2 domain belong to C2-PLD subclass; the PLDs with phox homology (PX) or pleckstrin homology (PH) domains belong to PX/PH-PLD subclass; the PLDs possessing a signal peptide (SP) in place of the usual C2 or PX/PH domains belong to SP-PLD subclass (Tang et al., 2016b). According to sequence characteristics and biochemical properties, the PLD members can be further subdivided into different isoforms, including PLDαs, PLDβs, PLDγs, PLDδs, PLDεs, PLDζs, and/or PLDφs. The isoforms α, β, γ, δ and ε are part of C2-PLDs; ζ and φ isoforms are attached to PX/PH-PLDs and SP-PLDs, respectively (Yao et al., 2021).

The different PLD isoforms are found to have specific subcellular localizations, lipid selectivity and reaction requirements, which lead to their unique cytological and biological functions in particular signaling pathways (Wang, 2005). In *Arabidopsis*, the phenotypic changes caused by the absence of one *PLD* member cannot be offset by the other 11 *PLD*s (Hong et al., 2016). PLDα1 is the predominant PLD and has been proved to regulate drought and salt tolerance by stimulating the accumulation of abscisic acid (ABA) and jasmonic acid (JA) (Li et al., 2009). The repressed expression of *PLDα1* may result in a reduced sensitivity to ABA and drought-induced stomatal closure (Sang et al., 2001). The *PLDα1* knock-out mutants display increased sensitivities to salinity and water deficiency and tend to induce ABA-responsive genes more readily, whereas the overexpression of *PLDα1* have decreased sensitivities (Bargmann et al., 2009). PLDδ is a signal enzyme that can connect microtubules with plasma membrane. The expression levels of *PLDδ* significantly increase under dehydration, salinity, and ABA treatments (Angelini et al., 2018). The knockout of *PLDδ* makes plants sensitive to freezing, heat and oxidative stresses (Li et al., 2004; Liu et al., 2021; Song et al., 2021). PLDζs are similar to animal PLDs in structure and have unique functions in root growth. PLDζ2-derived PA can promote root hair development under phosphorus deficiency by suppressing the vacuolar degradation of auxin efflux carrier PIN-FORMED2 (Lin et al., 2020). PLDζ1 shows crucial roles in both ionic and osmotic stress-induced auxin carrier dynamics during salt stress (Korver et al., 2020). Overall, most of the knowledge about *PLDs* and PLD-mediated lipid signaling has been revealed from studies on model plant *Arabidopsis* and some other crops. But the information about peanut *PLDs* and their roles in regulating developmental features and abiotic stresses yet need to be studied.

Cultivated peanut is a classic natural allotetraploid (AABB, 2n=4x=40). It arose from the interspecific hybridization and subsequent chromosome doubling of two diploid species *Arachis duranensis* (AA, 2n=2x=20) and *Arachis ipaensis* (BB, 2n=2x=20). Recently, the genomes of *A. duranensis*, *A*. *ipaensis* and *A*. *hypogaea* have been completely sequenced, which open a new chapter of *Arachis* genomic studies (Bertioli et al., 2016; Bertioli et al., 2019). In present study, we identified 90 *PLD* genes in these three *Arachis* species and evaluated their sequence characteristics, gene structures, conserved domains, phylogenetic relationships, expansion patterns, physiochemical properties of proteins, *cis*-regulatory elements, protein-protein interactions, miRNA-genes regulatory networks and expression profiles in different tissues and multiple abiotic stresses. These fundings may provide a comprehensive characterization of *Arachis PLDs* and lay a theoretical basis for further functional analysis of *PLDs* in regulating abiotic stress tolerance in peanut.

## Materials and methods

### Identification of *PLD* gene families in peanut and its two progenitors

The genome data (version 1.0) of *A. duranensis*, *A*. *ipaensis* and *A*. *hypogaea* were downloaded from the PeanutBase database (https://www.peanutbase.org/peanut_genome). The protein and nucleotide sequences of *PLDs* in *Arabidopsis* (TAIR10), soybean (version 2.1) and cotton (version 2.0) were retrieved from the Ensemble database (http://plants.ensembl.org/index.html) and used as queries to perform BLASTP and BLASTN searches against the *Arachis* genome data. The Hidden Markov Model (HMM) profiles for *PLDs* (PF00614) were obtained from the Pfam (https://pfam.xfam.org/) and used to perform HMMER searches in the *Arachis* proteome database. The protein sequences identified by both above methods were integrated and parsed by manual editing to remove the redundant. The remaining were considered as candidate *Arachis* PLD proteins and finally submitted to the SMART (http://smart.embl-heidelberg.de/) and InterProScan (http://www.ebi.ac.uk/interpro/) to analyze the presence of characteristic and functional domains. The protein size (aa), molecular weight (Mw), theoretical isoelectric point (pI), instability index (II), aliphatic index (AI) and grand average of hydropathicity (GRAVY) of *Arachis* PLD proteins were calculated by the ExPASy (https://web.expasy.org/protparam/). The subcellular localization was predicted using the Plant-mPLoc server (http://www.csbio.sjtu.edu.cn/).

### Phylogenetic analysis, chromosomal localization and gene nomenclature

The multiple sequence alignment of non-redundant PLD protein sequences in *A. duranensis*, *A*. *ipaensis*, *A*. *hypogaea*, *Arabidopsis*, soybean and cotton was performed using the Clustal W with the default settings (Thompson et al., 1994). A phylogenetic tree was constructed using the neighbor joining (NJ) method in MEGA7 software with following parameters: P-distance, pairwise gap deletion and bootstrap with 1000 replicates (Kumar et al., 2016).

The information regarding the detailed positions of *Arachis PLD*s on chromosomes were obtained from the *Arachis* genome database and visualized using the MapChart 2.32 software (Voorrips, 2002). The nomenclature of *Arachis PLD* genes was based on the results of phylogenetic analysis and chromosomal localization. Identified *PLD* genes in *A. duranensis*, *A*. *ipaensis* and *A*. *hypogaea* were named as *AdPLD*, *AiPLD* and *AhPLD* followed by roman letters, numbers and capital letters corresponding to their respective orthologs and chromosomal coordinates.

### Gene structure, conserved domain and protein motifs

The exon-intron organization of *Arachis PLD* genes was displayed using the Gene Structure Display Server (GSDS2.0) (http://gsds.cbi.pku.edu.cn/) by comparing their coding sequences (CDS) and corresponding genomic sequences. The conserved domains of *Arachis* PLD proteins were identify using the Pfam and SMART tools with the default cut off parameters. The conserved motifs of *Arachis* PLD proteins were investigated by the online tool Multiple Expectation maximization for Motif Elicitation (MEME) (https://meme-suite.org/meme/tools/meme) with following parameters: the maximum motif number was 30; the minimum motif width was 6; and the maximum motif width was 50. The identified protein motifs were further annotated with InterProScan.

### Gene duplication events and adaptive evolution analysis

The homologous *PLD* gene pairs were identified based on the results of chromosomal localization, multiple sequence alignments and phylogenetic analysis. The adopted criteria for gene duplication events were that the shared aligned sequence covered over 70% of the longer sequence and the minimum similarity of aligned regions was 70% (Tang et al., 2016b). The tandem duplications have been characterized as multiple members of one gene family occurring within the same or neighboring intergenic regions. The segmental duplications have been defined as homologous genes that result from large-scale events, such as whole genome duplications or large chromosomal region duplications. The duplication events and collinear relationships of *Arachis PLDs* were analyzed using the MCScanX and MCScanX-transposed toolkits (Wang et al., 2013) and visualized by the Circos software (Krzywinski et al., 2009).

The non-synonymous substitution rate (*Ka*) and synonymous substitution rate (*Ks*) of the duplicated gene pairs were calculated to assess the molecular selection effect using the *KaKs*_Calculator 2.0 software (Wang et al., 2010). The gene pairs with the *Ka* and *Ks* value of 0 as well as the *Ks* value more than 2 were discarded, as they might result from the sequence saturation or misalignment. The *Ka*/*Ks* ratio was then calculated to show the selection pressure for duplicated *PLD* genes. The *Ka*/*Ks* ratio >1, <1 or =1 represented positive, negative (purifying selection) and neutral evolution, respectively. The divergence time of *AhPLD* gene pairs was estimated by the formula T= Ks/2r, where r indicates the neutral substitution rate (r = 8.12 × 10^-9^ Ks yr^−1^) (Bertioli et al., 2016).

### *Cis*-regulatory elements prediction and interaction networks analysis

The 2000 base pair (bp) DNA sequences in upstream regions of *AhPLD*s were extracted from the peanut genome database and submitted to the PlantCare (http://bioinformatice.psb.ugent.be/webtools/plantcare/) for the identification of putative *cis*-regulatory elements. The results were finally visualized by the TBtools software (Chen et al., 2020).

The STRING database (https://string-db.org/) was used to analyze the interaction relationships between AhPLDs and other proteins with the confidence parameter set at the threshold of 0.4. The well-characterized plant *Arabidopsis* was as the query organism. The predicted protein-protein interaction (PPI) network was displayed by the Cytoscape 3.7.2 software (Shannon et al., 2003).

The targeting relationship between *AhPLDs* and microRNA (miRNA) were predicated using the psRNATarget Server (http://plantgrn.noble.org/psRNATarget/) with the expectation score of 3.5 (Rizwan et al., 2022b). The peanut miRNA sequences were obtained from the miRbase database (http://www.mirbase.org/). The cDNA sequences of *AhPLDs* were extracted as the candidate targets. The linkage of the predicted miRNAs and corresponding target genes were displayed by the Cytoscape 3.7.2 software (Shannon et al., 2003).

### Expression profiles of *AhPLDs* in different tissues based on RNA-sequencing analysis

The RNA-sequencing (RNA-seq) data of 22 peanut tissues at vegetative, reproductive and seed development stages were retrieved from the National Center for Biotechnology Information BioProject (https://www.ncbi.nlm.nih.gov/bioproject/) with the accession number PRJNA291488. The fragments per kilobase of exon model per million mapped reads (FPKM) method in Cufflinks software (http://cufflnks.cbcb.umd.edu/) were used to calculate the transcript abundance. The log_2_FPKM values were displayed in the form of heatmaps by the HemI software (Deng et al., 2014).

### Plant materials, growth conditions and stress treatments

The allotetraploid peanut (*A*. *hypogaea* cultivar ‘Nonghua 5’) planted in large areas of northeast China was used as experimental materials in this study. The seeds were surface sterilized with 3% sodium hypochlorite, and washed five times with distilled water, and kept in dark to germinate. The germinated seeds were sown in round plastic pots filled with clean sandy soil and grown in a climate chamber with a 16 h light (28 °C)/8 h dark (23 °C) cycle, a photosynthetic photon flux density of 400 µmol m^−2^ s^−1^, and a relative humidity of 70%. After 14 days, the three-leaf seedlings were transferred from sandy soil into hydroponic cultures and grown for 3 days to recover before initiating any stress treatments. For drought and salt stresses, seedlings were incubated in 20% (w/v) polyethylene glycol (PEG-6000) and 250mM NaCl solution, respectively. For cold stress, the temperature of the climate chamber was reduced to 6°C without changing other growth conditions. The second leaves were collected at 0, 6, 12, 24 and 48 h of each treatment with three biological replicates, and frozen in liquid nitrogen immediately and stored at -80°C.

### RNA extraction and expression analysis by quantitative real-time RT-PCR

The total RNA for each sample was extracted by TRIzol reagent (Carlsbad, CA, USA) according to the manufacturer’s protocol. The RNA concentration was tested using a micro-spectrophotometer (OD260/280). The RNA integrity was tested using the Agilent Bioanalyzer 2100 system. 1 μg of total RNA was used to synthesize first strand cDNA by Takara Reverse Transcription System (TaKaRa, Shuzo, Otsu, Japan). The expression profiles of *AhPLDs* under various abiotic stresses was detected by quantitative real-time PCR (qRT-PCR) using the specific primers as listed in **Table S1**. The real-time quantification was performed with SYBR Premix Ex Taq™ (TaKaRa, Shuzo, Otsu, Japan) according to the manufacturer’s instructions. PCR mixtures (10 µL) contained 1.0 µL cDNA, 0.3 µL each primer, 3.4 µL ddH_2_O and 5.0 µL SYBRfi Green Master Mix. The amplification conditions were as follows: 60 s denaturation at 95°C, followed by 40 cycles of 95°C for 15 s, 55°C for 30 s, and 72°C for 60 s. Three biological replicates per sample and three technical replicates per biological replicate were used for the analysis. The *AhACT11* was served as the internal reference gene, and the relative expression values were calculated using the 2^-ΔΔct^ method (Chi et al., 2012).

## Results

### Genome-wide identification of *PLD* genes in peanut and its two progenitors

**Figure 1:**
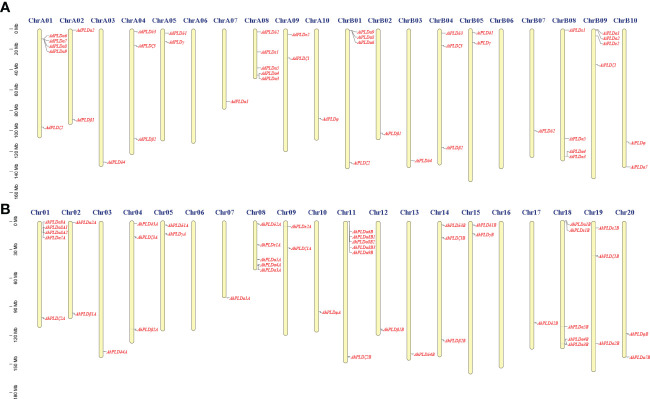
Chromosomal distribution of *Arachis PLD* genes. **(A)** Chromosomal distribution of *PLD* genes in *A*. *duranensis* and *A*. *ipaensis*. **(B)** Chromosomal distribution of *PLD* genes in *A*. *hypogaea*. Yellow color bars represent the chromosomes, and the location of *PLD* genes has been marked alongside.

Using the method as described above, a total of 22 *AdPLD*s, 22 *AiPLD*s and 46 *AhPLD*s were identified in *A. duranensis*, *A*. *ipaensis* and *A*. *hypogaea* genomes, respectively (**Table S2**). Chromosome localization found that the identified *AdPLD*s and *AiPLD*s were unevenly distributed in nine out of ten chromosomes of *A. duranensis* (A genome) and *A*. *ipaensis* (B genome), respectively (**Figure 1A**). Among them, A01 and A08 chromosomes possessed the most abundant *PLDs* with each containing five *AdPLD*s, followed by B01, B08 and B09 chromosomes with each containing four *AiPLD*s, but no *PLDs* was found on chromosomes A06 and B06. In *A*. *hypogaea*, 46 *AhPLD*s were located on 18 out of 20 chromosomes (**Figure 1B**), of which chromosomes 01, 08, 11 and 18 contained the most *AhPLD*s but chromosomes 06 and 16 had no *AhPLD*.

Furthermore, the sequence characteristics of PLD proteins in three *Arachis* species were analyzed (**Table 1**). The opening reading frame (ORF) lengths of all the 90 *PLDs* ranged from 1305 bp to 3417 bp, which encoded polypeptides of 507 aa to 1138 aa with predicted MWs ranging from 57.9 kD to 126.94 kD and theoretical p*I*s ranging from 5.16 to 8.44. According to the II values, 49 *Arachis* PLDs were considered as the stable proteins (II<40), and 41 *Arachis* PLDs belonged to the unstable proteins (II>40). Besides, all *Arachis* PLD proteins had high AI values from 69.47 to 86.41 but minus GRAVY values from -0.179 to -0.756, indicating they were stable over a wide temperature range and were hydrophilic and highly soluble in water. Subcellular localization prediction revealed that most *Arachis* PLD proteins were in the cytoplasm, endoplasmic reticulum and vacuole, only a few were in the chloroplast, nucleus and plasma membrane.

**Table 1:** Detailed information of PLD genes identified in *A. duranensis*, *A*. *ipaensis* and *A*. *hypogaea*.

Gene name	PeanutBase ID	Chr	ORF length (bp)	Deduced protein						Subcellular location
				Size (aa)	MW (kDa)	pI	II	AI	GRAVY	
AdPLDα1	Aradu.4Q29Q.1	A07	2292	763	86.76	5.74	40.87	82.66	-0.380	Endoplasmic reticulum; Vacuole
AdPLDα2	Aradu.FS7LG.1	A02	2325	774	87.90	6.20	43.62	80.74	-0.402	Endoplasmic reticulum; Vacuole
AdPLDα3	Aradu.H7I4I.1	A08	2448	815	93.29	6.19	37.99	81.47	-0.485	Endoplasmic reticulum; Vacuole
AdPLDα4	Aradu.H7I4I.2	A08	2469	822	94.43	6.37	35.86	80.47	-0.432	Endoplasmic reticulum; Vacuole
AdPLDα5	Aradu.B9ITB.1	A08	2454	817	93.36	6.22	41.63	84.35	-0.373	Endoplasmic reticulum; Vacuole
AdPLDα6	Aradu.ZT8PK.1	A01	2103	700	81.03	5.21	42.81	69.47	-0.756	Cytoplasm; Nucleus
AdPLDα7	Aradu.PE7A6.1	A01	2388	795	91.26	8.44	38.26	79.55	-0.479	Cytoplasm
AdPLDα8	Aradu.SYE4G.1	A01	2400	799	91.30	6.25	37.42	83.43	-0.448	Cytoplasm
AdPLDα9	Aradu.D9A5M.1	A01	1305	693	78.89	6.67	35.49	82.87	-0.392	Cytoplasm
AdPLDβ1	Aradu.7N75D.1	A02	3417	1138	126.58	8.30	52.61	73.76	-0.447	Chloroplast; Nucleus
AdPLDβ2	Aradu.NT5AR.1	A04	3351	1116	124.57	7.03	46.39	72.31	-0.476	Chloroplast; Nucleus
AdPLDγ	Aradu.X0Z7E.1	A05	2550	849	95.66	6.68	32.27	80.45	-0.409	Cytoplasm
AdPLDδ1	Aradu.UM7P3.1	A05	2592	863	98.18	6.62	34.59	76.95	-0.424	Cytoplasm
AdPLDδ2	Aradu.FN19Y.1	A08	1524	507	57.90	6.58	36.63	78.24	-0.407	Cytoplasm
AdPLDδ3	Aradu.401JR.1	A04	2544	847	95.84	6.59	32.70	83.21	-0.369	Cytoplasm
AdPLDδ4	Aradu.J7XF6.1	A03	2526	841	95.32	6.66	34.17	80.19	-0.394	Cytoplasm
AdPLDε1	Aradu.76V5M.1	A08	2292	763	87.69	6.25	35.22	78.09	-0.423	Cytoplasm
AdPLDε2	Aradu.AA5P6.1	A09	2325	774	88.47	6.15	39.94	78.89	-0.478	Cytoplasm
AdPLDζ1	Aradu.KSI0H.1	A09	3049	1015	115.78	6.38	41.52	86.41	-0.438	Cytoplasm
AdPLDζ2	Aradu.AF7PL.1	A01	3330	1109	126.46	6.53	43.17	82.38	-0.398	Cytoplasm
AdPLDζ3	Aradu.NR2Z3.1	A04	3354	1117	126.94	6.06	49.88	80.84	-0.407	Cytoplasm
AdPLDφ	Aradu.W0B0I.1	A10	1593	530	59.78	6.28	36.67	80.02	-0.179	Plasma membrane
AiPLDα1	Araip.YJB9F.1	B09	2355	784	89.36	5.65	41.53	83.05	-0.400	Endoplasmic reticulum; Vacuole
AiPLDα2	Araip.MYU90.1	B09	2424	807	91.60	6.14	44.57	80.22	-0.408	Endoplasmic reticulum; Vacuole
AiPLDα3	Araip.0C2UG.1	B08	2448	815	93.19	6.27	40.76	80.87	-0.490	Endoplasmic reticulum; Vacuole
AiPLDα4	Araip.0C2UG.2	B08	2136	711	81.35	5.72	38.08	81.66	-0.421	Endoplasmic reticulum; Vacuole
AiPLDα5	Araip.6R3VE.1	B08	2454	817	93.57	6.27	40.50	85.19	-0.363	Endoplasmic reticulum; Vacuole
AiPLDα6	Araip.03APC.2	B01	2394	797	91.57	6.28	46.77	76.21	-0.532	Cytoplasm; Nucleus
AiPLDα7	Araip.I4SWQ.1	B10	2439	812	93.02	6.44	43.50	80.60	-0.527	Cytoplasm
AiPLDα8	Araip.03APC.1	B01	2385	794	90.68	6.38	37.40	81.75	-0.456	Cytoplasm
AiPLDα9	Araip.72Y3Y.1	B01	2121	706	80.63	6.19	31.25	78.73	-0.541	Cytoplasm
AiPLDβ1	Araip.10YD3.1	B02	3312	1103	122.24	8.14	53.50	71.70	-0.499	Chloroplast; Nucleus
AiPLDβ2	Araip.CEE4L.1	B04	3351	1116	124.53	7.02	45.51	72.75	-0.467	Chloroplast; Nucleus
AiPLDγ	Araip.67DP4.1	B05	2550	894	95.54	6.83	31.68	81.59	-0.385	Cytoplasm
AiPLDδ1	Araip.5S0ML.1	B05	2592	863	98.11	6.54	34.67	77.18	-0.410	Cytoplasm
AiPLDδ2	Araip.Z1PQN.1	B07	2547	848	96.41	6.58	37.50	79.65	-0.370	Cytoplasm
AiPLDδ3	Araip.SC81T.1	B04	2544	847	95.93	6.61	32.03	83.32	-0.373	Cytoplasm
AiPLDδ4	Araip.N118V.1	B03	2526	841	95.12	6.73	34.88	80.19	-0.395	Cytoplasm
AiPLDε1	Araip.ZU18G.1	B08	2292	763	87.65	6.36	35.66	76.42	-0.431	Cytoplasm
AiPLDε2	Araip.F3NWG.1	B09	2816	938	106.91	5.91	43.06	78.82	-0.484	Cytoplasm
AiPLDζ1	Araip.QK1BG.1	B09	3237	1078	122.20	6.34	43.03	85.53	-0.435	Cytoplasm
AiPLDζ2	Araip.C34P5.1	B01	2676	891	101.55	7.05	41.19	81.40	-0.408	Cytoplasm
AiPLDζ3	Araip.3PC35.1	B04	2949	982	111.65	6.02	49.11	83.30	-0.388	Cytoplasm
AiPLDφ	Araip.37594.1	B10	1593	530	59.92	6.40	35.75	80.19	-0.198	Plasma membrane
AhPLDα1A	Arahy.PYKR9B.1	07	2424	807	91.88	5.54	42.32	83.82	-0.391	Endoplasmic reticulum; Vacuole
AhPLDα1B	Arahy.JUK473.1	18	2424	807	91.88	5.54	42.32	83.82	-0.391	Endoplasmic reticulum; Vacuole
AhPLDα2A	Arahy.019LBF.1	02	2424	807	91.62	6.11	44.47	79.62	-0.408	Endoplasmic reticulum; Vacuole
AhPLDα2B	Arahy.7PV95K.1	19	2424	807	91.60	6.14	44.57	80.22	-0.408	Endoplasmic reticulum; Vacuole
AhPLDα3A	Arahy.GVK7JC.1	08	2448	815	93.26	6.19	38.21	80.87	-0.491	Endoplasmic reticulum; Vacuole
AhPLDα3B	Arahy.ES1PUL.1	18	2448	815	93.19	6.27	40.76	80.87	-0.490	Endoplasmic reticulum; Vacuole
AhPLDα4A	Arahy.A46I61.1	08	2469	822	93.74	6.21	37.28	82.62	-0.413	Endoplasmic reticulum; Vacuole
AhPLDα4B	Arahy.ES1PUL.2	18	2469	822	93.69	6.18	36.38	82.49	-0.405	Endoplasmic reticulum; Vacuole
AhPLDα5A	Arahy.ITK9EF.1	08	2454	817	93.39	6.22	41.54	84.35	-0.374	Endoplasmic reticulum; Vacuole
AhPLDα5B	Arahy.F6RD2F.1	18	2454	817	93.57	6.27	40.50	85.19	-0.363	Endoplasmic reticulum; Vacuole
AhPLDα6B	Arahy.JL8VMK.1	11	2532	843	97.16	5.16	52.00	72.98	-0.652	Cytoplasm; Nucleus
AhPLDα7A	Arahy.80NXVT.1	01	2388	795	91.26	8.44	38.26	79.55	-0.479	Cytoplasm
AhPLDα7B	Arahy.79BS07.1	20	2439	812	93.02	6.44	43.50	80.60	-0.527	Cytoplasm
AhPLDα8A1	Arahy.C52HL3.1	01	2400	799	91.50	6.23	36.04	82.47	-0.447	Cytoplasm
AhPLDα8A2	Arahy.F67ZD9.1	01	2400	799	91.19	6.25	37.41	83.30	-0.445	Cytoplasm
AhPLDα8B1	Arahy.LN5JTA.1	11	2385	794	90.64	6.25	35.28	82.12	-0.443	Cytoplasm
AhPLDα8B2	Arahy.19WSGG.1	11	2385	794	90.76	6.38	37.87	82.62	-0.443	Cytoplasm
AhPLDα8B3	Arahy.6051AB.1	11	2382	793	90.91	6.17	36.78	80.87	-0.468	Cytoplasm
AhPLDα9A	Arahy.KC14LW.1	01	2061	686	77.51	7.31	34.09	82.87	-0.378	Cytoplasm
AhPLDα9B	Arahy.0K14UN.1	11	2121	706	80.63	6.19	31.25	78.73	-0.541	Cytoplasm
AhPLDβ1A	Arahy.3G00JX.1	02	3312	1103	122.33	8.13	53.27	72.31	-0.492	Chloroplast; Nucleus
AhPLDβ1B	Arahy.ZGV9T5.1	12	3312	1103	122.24	8.14	53.50	71.70	-0.499	Chloroplast; Nucleus
AhPLDβ2A	Arahy.Z6D9E8.1	04	3351	1116	124.60	7.03	46.14	72.40	-0.471	Chloroplast; Nucleus
AhPLDβ2B	Arahy.IIM5IQ.1	14	3351	1116	124.53	7.02	45.51	72.75	-0.467	Chloroplast; Nucleus
AhPLDγA	Arahy.NAJ0WW.1	05	2550	849	95.63	6.60	33.72	81.59	-0.393	Cytoplasm
AhPLDγB	Arahy.GAK0W0.1	15	2550	849	95.54	6.83	31.68	81.59	-0.385	Cytoplasm
AhPLDδ1A	Arahy.UU4P1L.1	05	2592	863	98.19	6.62	34.59	76.95	-0.424	Cytoplasm
AhPLDδ1B	Arahy.PYQ6LW.1	15	2592	863	98.18	6.62	34.59	76.95	-0.424	Cytoplasm
AhPLDδ2A	Arahy.J2YZ53.1	08	2547	848	96.38	6.73	37.27	80.09	-0.358	Cytoplasm
AhPLDδ2B	Arahy.I9WKA5.1	17	2547	848	96.41	6.58	37.50	79.65	-0.370	Cytoplasm
AhPLDδ3A	Arahy.NJG8XE.1	04	2544	847	95.84	6.59	32.70	83.21	-0.369	Cytoplasm
AhPLDδ3B	Arahy.XJ1GJM.1	14	2580	859	97.41	6.61	32.95	82.72	-0.369	Cytoplasm
AhPLDδ4A	Arahy.87HDGW.1	03	2529	842	95.33	6.66	34.34	80.10	-0.397	Cytoplasm
AhPLDδ4B	Arahy.UD7LPN.1	13	2526	841	95.12	6.73	34.88	80.19	-0.395	Cytoplasm
AhPLDε1A	Arahy.PTFL6P.1	08	2292	763	87.73	6.27	34.83	77.44	-0.434	Cytoplasm
AhPLDε1B	Arahy.SE7ST2.1	18	2292	763	87.65	6.36	35.66	76.42	-0.431	Cytoplasm
AhPLDε2A	Arahy.1L37A5.1	09	2427	808	92.19	6.25	39.46	76.88	-0.509	Cytoplasm
AhPLDε2B	Arahy.63QAK2.1	19	2433	810	92.57	6.14	41.40	76.33	-0.530	Cytoplasm
AhPLDζ1A	Arahy.T1UA0C.1	09	3231	1076	122.63	6.21	43.65	85.50	-0.443	Cytoplasm
AhPLDζ1B	Arahy.Y1617F.1	19	3231	1076	122.48	6.14	43.42	84.52	-0.446	Cytoplasm
AhPLDζ2A	Arahy.HZZY15.1	01	2676	891	101.72	7.19	41.48	81.40	-0.409	Cytoplasm
AhPLDζ2B	Arahy.M1WUGI.1	11	2676	891	101.55	7.05	41.19	81.40	-0.408	Cytoplasm
AhPLDζ3A	Arahy.I1DXP3.1	04	3354	1117	126.94	6.06	49.88	80.84	-0.407	Cytoplasm
AhPLDζ3B	Arahy.4IL771.1	14	3099	1032	117.75	6.11	48.71	83.04	-0.396	Cytoplasm
AhPLDφA	Arahy.XZ9AUI.1	10	1593	530	59.84	6.40	37.50	80.02	-0.192	Plasma membrane
AhPLDφB	Arahy.G1SIKM.1	20	1593	530	59.92	6.40	35.75	80.19	-0.198	Plasma membrane

### Phylogenetic analysis, gene structure and conserved motifs of *Arachis PLD* genes

**Figure 2:**
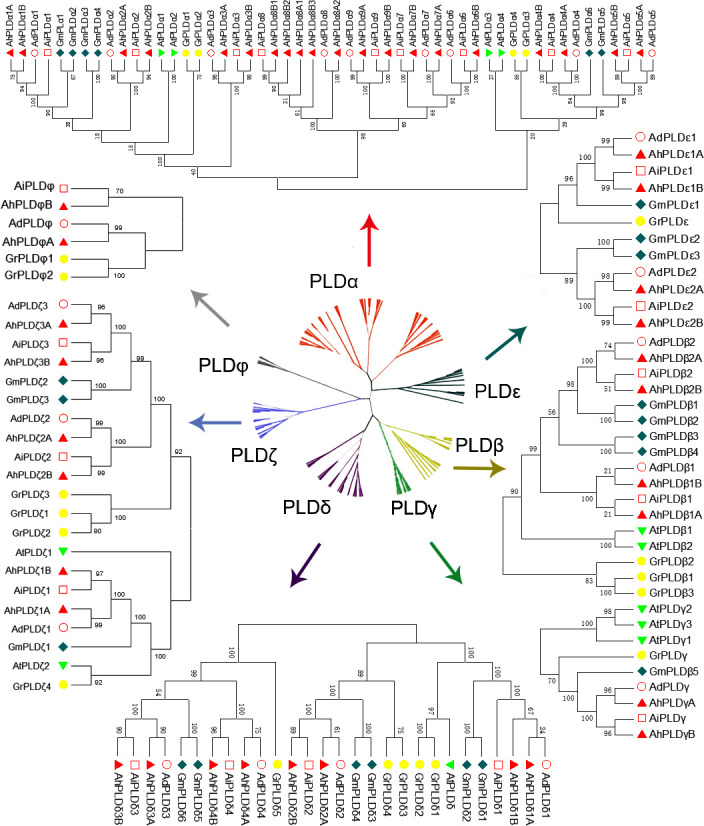
Phylogenetic tree of *PLD* gene family with bootstrap values among three *Arachis* species, *Arabidopsis*, soybean and cotton. The red, yellow, green, purple, dark green, blue and gray branches represent α, β, γ, δ, ε, ζ and ϕ isoforms, respectively. The red circles, red squares, red triangles, green triangles, dark green diamonds and yellow circles represent the proteins of *A. duranensis* (Ad), *A. ipaensis* (Ai), *A. hypogaea* (Ah), *Arabidopsis* (At), soybean (Gm) and cotton (Gr), respectively.

To explore the phylogenetic relationships and evolutionary patterns of the *PLDs* in peanut and its two progenitors, a neighbor-joining tree was constructed using the full-length protein sequence alignments of 90 *Arachis* PLDs, 12 AtPLDs, 23 GmPLDs and 20 GrPLDs. All the PLDs from different plant species were clearly divided into seven well-supported sub-clades based on the similarity with cotton PLDs, comprising α, β, γ, δ, ε, ζ and φ isoforms (**Figure 2**). Among them, the α constituted the largest clade containing 38 *Arachis* PLDs (20 AhPLDαs, nine AdPLDαs and AiPLDαs), the δ formed the second largest clade containing 16 *Arachis* PLDs (eight AhPLDδs, four AdPLDδs and AiPLDδs), and the φ was the smallest subgroup containing only four *Arachis* PLDs (two AhPLDφs, one AdPLDφs and AiPLDφs). Besides, the β and γ showed closely together and were not explicitly separated from each other. Within the separate clades, isoforms ε and φ were absent in *Arabidopsis*, isoforms γ and φ were absent in soybean, but all seven isoforms were present in *Arachis* and cotton. Obviously, *Arachis* PLDs showed closer to GmPLDs but more distant from AtPLDs. According to the phylogenetic relationships with orthologs in soybean, cotton and *Arabidopsis*, as well as the physical location on chromosomes, 22 AdPLDs and 22 AiPLDs were renamed as Ad/AiPLDα1-9, Ad/AiPLDβ1, Ad/AiPLDβ2, Ad/AiPLDγ, Ad/AiPLDδ1-4, Ad/AiPLDε1, Ad/AiPLDε2, Ad/AiPLDζ1-3 and Ad/AiPLDφ. The 46 AhPLDs were renamed as AhPLDα1A/1B-5A/5B, AhPLDα6B, AhPLDα7A/7B, AhPLDα8A1/8A2/8B1/8B2/8B3, AhPLDα9A/9B, AhPLDβ1A/1B, AhPLDβ2A/2B, AhPLDγA/B, AhPLDδ1A/1B-4A/4B, AhPLDε1A/1B, AhPLDε2A/2B, AhPLDζ1A/1B- 3A/3B and AhPLDφA/B.

**Figure 3:**
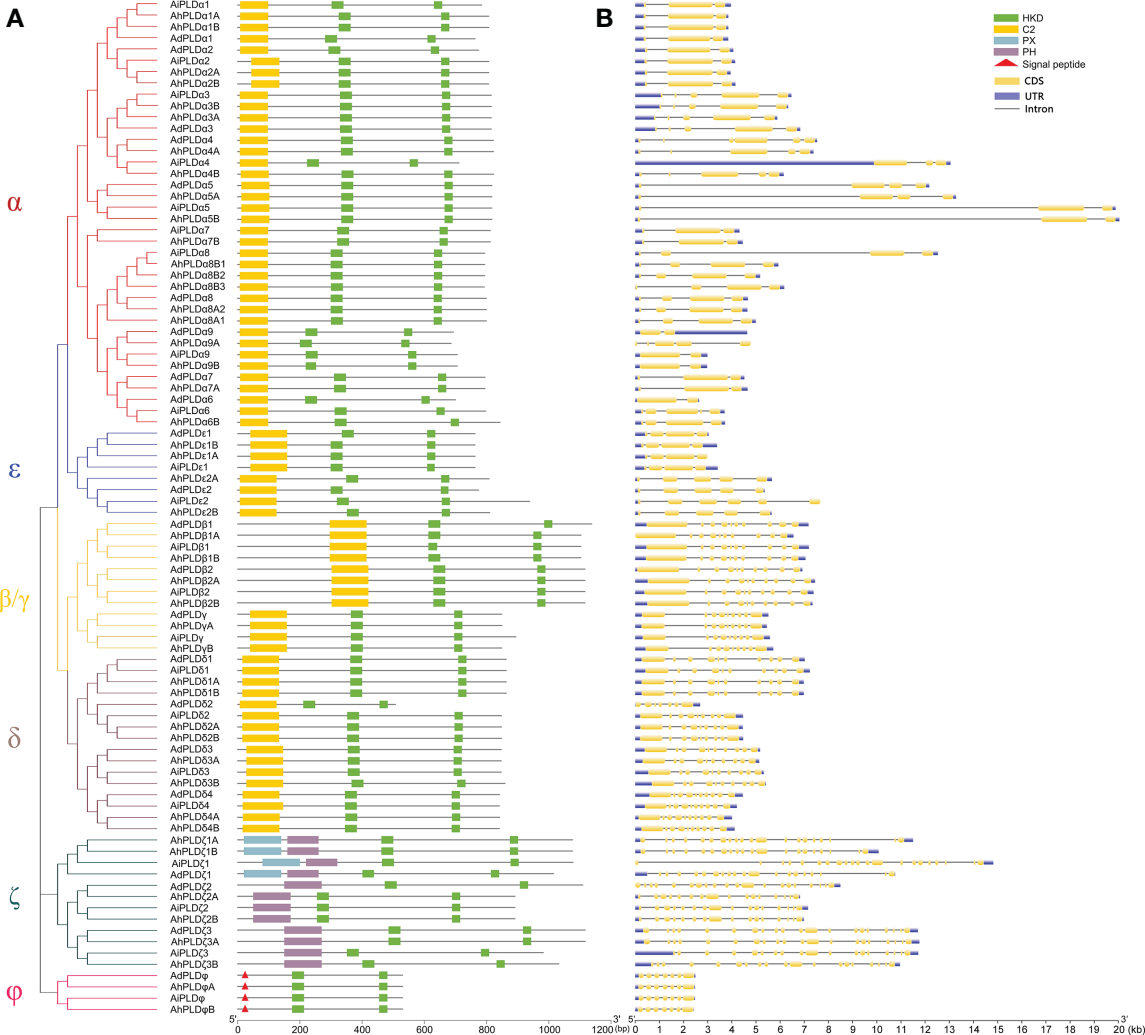
Phylogenetic relationship, conserved domains and gene structure of *PLD* genes in peanut and its two progenitors. **(A)** Schematic representation of the phylogenetic tree and conserved domain of *PLD* genes in three *Arachis* species. The red, blue, yellow, purple, dark green and pink branches on phylogenetic tree represent the α, ε, β/γ, δ, ζ and ϕ isoforms, respectively. The yellow, green, blue and purple rectangles represent the C2, HKD, PX and PH domains, respectively. The red triangles represent the signal peptides. **(B)** Exon-intron structure of *PLD* genes in three *Arachis* species. The blue boxes represent exons. The yellow boxes represent coding sequences. The black lines represent introns.

To confirm the authenticity and integrity of identified *Arachis PLD* genes, their functional domains were analyzed by searching the Pfam and SMART databases. As expected, all *Arachis* PLD proteins possessed two structurally conserved HKD domains at C-terminal and were divided into three subgroups (**Figure 3A**). Isoforms α, β, γ, δ and ε contained C2 domain at the N-terminal and belonged to the C2-PLDs. PLDζs contained PX and/or PH domain at the N-terminal and belonged to the PX/PH-PLDs. PLDφs contained signal peptide at the N-terminal instead of C2 or PX/PH domain and belonged to the SP-PLDs. Furthermore, the distribution of exons and introns within each PLD protein was also analyzed. As shown in **Figure 3B**, all the 90 *Arachis* PLDs were comprised of multiple exons and introns. The number of introns determined for *Arachis* PLDs ranged from one in AdPLDα6, AdPLDα9, AiPLDα9 and AhPLDα9B to 20 in AiPLDζ1. The members of all *Arachis* PLD isoforms showed similar exon-intron structure withing their respective subgroups. It was consistent with the phylogenetic classification depicted in the left panel of **Figure 3A**. For instance, both α and ε subgroups included members with one to five introns, the members in β/γ and δ subgroups possessed eight or nine introns (except AdPLDδ2 that had six introns), the members in ζ subgroup contained 15 to 20 introns, and all six members in φ subgroup had six introns.

**Figure 4:**
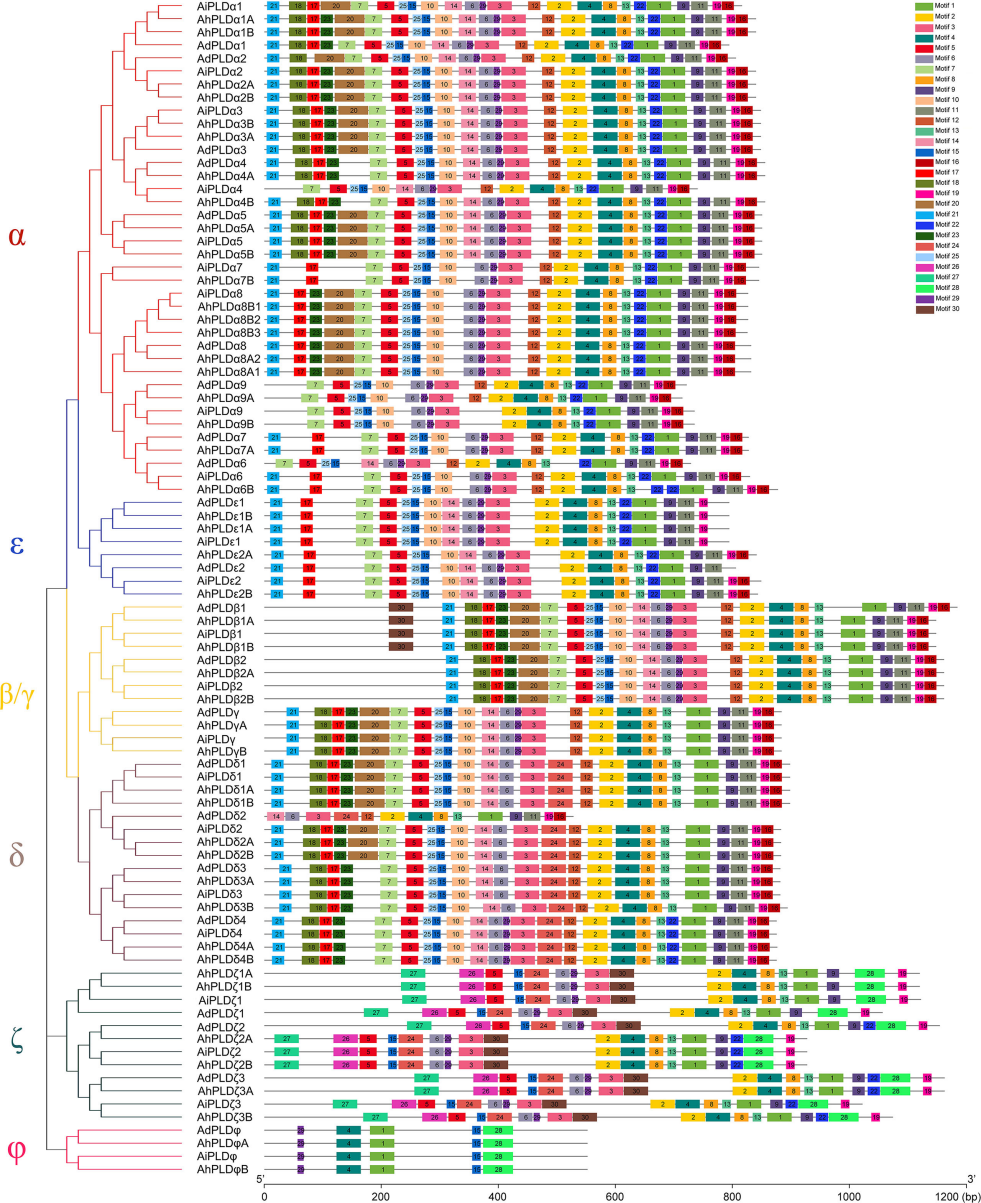
Motif composition of PLD proteins in peanut and its two progenitors. The red, blue, yellow, purple, dark green and pink branches on phylogenetic tree represent the α, ε, β/γ, δ, ζ and ϕ isoforms, respectively. The motifs, numbers 1-30, are displayed in different colored boxes.

To reveal the typical domain characteristics of *Arachis* PLD subgroups, the conservation of amino acid residues in functional domains were analyzed based on the alignments of these PLDs. A total of 30 distinct motifs designated as Motif 1 to Motif 30 were identified (**Figures 4**, **S1**; **Table S3**). The members within the same subfamilies were usually shared similar motif composition, for example, C2-PLDs possessed 17 to 25 motifs, of which Motif 1-11, Motif 13, Motif 15 and Motif 25 were highly conserved; PX/PH-PLDs possessed 18 motifs, including Motif 1-9, Motif 13, Motif 15, Motif 19, Motif 24 and Motif 26-30; whereas SP-PLDs only contained five motifs, including Motif 1, Motif 4, Motif 15, Motif 28 and Motif 29. By comparison, the Motif 26 and Motif 27 were only present in the C-terminus of PX/PH-PLDs but absent in C2-PLDs and SP-PLDs, suggesting these two motifs were specific to the PX and PH domains. Moreover, almost all these motifs could be matched to the annotated motifs in InterProScan and were functionally associated with PLD activity. The Motif 1, Motif 6, Motif 14 and Motif 29 were annotated as the conserved HKD domains (HxKxxxxD), especially the Motif 1 was uniformly observed in all *Arachis* PLDs. The Motif 4, also observed in all PLD proteins, contained a core triplet of amino acids “ERF” followed by a highly conserved hydrophobic region “VYVVV”. The Motif 2 contained a regular-expression sequence “IYIENQ[F/Y]F”, of which the seventh amino acid, Phenylalanine (F), appeared in all PX/PH-PLDs but was often substituted by the Tyrosine (Y) in C2-PLDs. The Motif 3 contained the conserved amino acid”xxGPRxPWHDxHxxxxGPAxxDVLTNFExRWRKxGx”, which was considered as the binding sites of PIP2.

### Gene duplication events and molecular evolution of *Arachis PLD* genes

**Figure 5:**
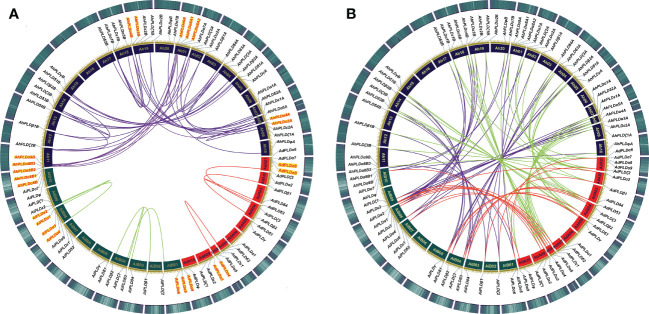
Syntenic relationships among *PLD* genes in peanut and its two progenitors. **(A)** Syntenic relationships of *PLD* genes within *A*. *duranensis*, *A*. *ipaensis* and *A*. *hypogaea*, respectively. **(B)** Syntenic relationships of *PLD* genes between *A*. *duranensis*, *A*. *ipaensis* and *A*. *hypogaea*. The chromosomes of *A*. *duranensis*, *A*. *ipaensis* and *A*. *hypogaea* were shown with red, dark green and dark purple colors, respectively. The putative homologous *PLD* genes are linked by different colored lines. The tandem duplicated *PLD* genes are marked with red font next to the chromosomes.

To clarify the roles of gene duplications in the expansion of *Arachis PLD* gene families, the synteny analysis including tandem duplication and whole genome duplication (WGD)/segmental duplication was performed based on the multiple and pairwise alignments of *Arachis PLDs*. As a result, a total of 131 orthologous/paralogous gene pairs were identified, of which 54 pairs were predicted to form paralogous gene pairs within the *A. duranensis*, *A. ipaensis* and *A. hypogaea* genomes, and nine pairs underwent the tandem duplications (*AdPLDα3-AdPLDα4*, *AdPLDα8-AdPLDα9*, *AiPLDα1-AiPLDα2*, *AiPLDα3-AiPLDα4*, *AiPLDα6-AiPLDα8-AiPLDα9*, *AhPLDα3A-AhPLDα4A*, *AhPLDα3B-AhPLDα4B*, *AhPLDα8A1-AhPLDα8A2-AhPLDα9A*, *AhPLDα6B-AhPLDα8B1-AhPLDα8B2-AhPLDα8B3-AhPLDα9B*) (**Figure 5A**; **Table S4**). Besides, 25, 28 and 24 segmental duplications were found in groups *AhPLDs-AdPLDs*, *AhPLDs-AiPLDs* and *AdPLDs-AiPLDs* respectively, but one group only contained *AiPLDα6* homoeologous gene (*AhPLDα6B*), suggesting the corresponding *AhPLDα6A* had lost during peanut evolution (**Figure 5B**; **Table S4**). Consequently, we presumed that the putative gene duplication events were main causes of *Arachis PLD* gene family expansion, and homoeologous gene pairs were generally raised from tandem or WGD/segmental duplication before polyploidization involved in evolution process.

To investigate which type of selection pressure had been involved in *PLD* gene divergence after duplication, the *Ka*/*Ks* ratios of duplicated *Arachis PLD* gene pairs were calculated on the basis of coding sequences (**Table S5**). The resulting pairwise comparison data showed that most homoeologous gene pairs had *Ka*/*Ks* ratios of < 1, indicating these gene pairs might have undergone strong purifying selection pressure with limited functional divergence that occurred after tandem or WGD/segmental duplication. Only three gene pairs (*AhPLDα8A2*-*AdPLDα8*, *AhPLDδ3B*-*AiPLDδ3* and *AhPLDδ3A*-*AhPLDδ3B*) had *Ka*/*Ks* ratios of > 1, suggesting these gene pairs might have experienced relatively rapid evolution following duplication. Meanwhile, according to Ks values, the divergence time of duplicated *Arachis PLD* gene pairs were estimated. As a result, these tandem and segmental duplications may occur in 3.02-70.62 and 0.10-119.83 Mya, respectively (**Table S5**).

### *Cis*-regulatory elements prediction in *AhPLDs* promotors

**Figure 6:**
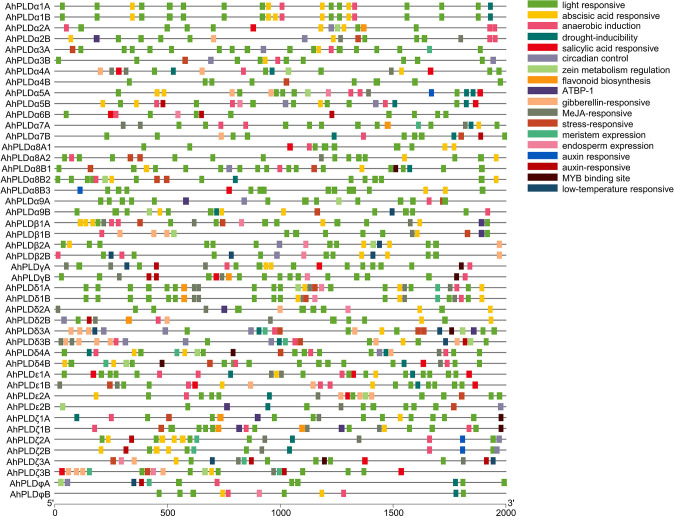
*Cis*-regulatory elements in the promoters of peanut *PLD* genes. Various *cis*-regulatory elements are displayed in different colored boxes.

To better understand the transcriptional regulation and potential functions of peanut *PLD* genes, the *cis*-regulatory elements present in promoters (2000 bp of 5’ upstream regions) of *AhPLD* genes were identified. Totally, 39 functional *cis*-elements related to growth and development, plant hormones and stress responses were obtained from 46 *AhPLD*s (**Table S6**). All *AhPLD* promoters had variable number of *cis*-regulatory elements and most of them were present in multiples (**Figure 6**). Among the 25 growth and development-related elements, light-responsive elements (87.59%), such as Box 4, G-box, GT1-motif, TCT-motif and GATA-motif, accounted for the most and widely distributed in all *AhPLD* promoters; others mainly included the circadian element (3.28%) related to circadian control, the O_2_-site (2.57%) related to zein metabolism regulation, the CAT-box (2.00%) related to meristem expression and the GCN4_motif (1.85%) related to endosperm expression.

The nine hormone-responsive *cis*-elements were related to five plant hormones. Among them, abscisic acid responsiveness element (35.84%, ABRE) was identified in 39 *AhPLD*s; MeJA-responsive elements (37.99%, CGTCA-motif/TGACG-motif) were identified in 27 *AhPLD*s; gibberellin-responsive elements (12.19%, GARE-motif/P-box/TATC-box) were identified in 17 *AhPLD*s; salicylic acid-responsive element (7.17%, TCA-element) was identified in 15 *AhPLD*s; auxin-responsive elements (6.81%, TGA-element/AuxRR-core) were identified in 14 *AhPLD*s (**Figure 6**; **Table S6**). There were three *AhPLD*s (*AhPLDα5A*, *AhPLDζ3A* and *AhPLDζ3B*) contained the five hormone-responsive elements above-mentioned, but *AhPLDα9A* did not contain any hormone-responsive elements. Besides, the promoter regions of eight *AhPLD*s (*AhPLDα1A*, *AhPLDα1B*, *AhPLDα3A*, *AhPLDα3B*, *AhPLDα8B1*, *AhPLDα8B2*, *AhPLDα9B* and *AhPLDφB*) only contained ABA-responsive elements, whereas *AhPLDε2B* and *AhPLDφA* only contained MeJA- and auxin- responsive elements, respectively. However, the ethylene-responsive *cis*-elements were not found in any *AhPLD* promoters.

Moreover, there were five *cis*-elements found to be responsive to various stresses, including anaerobic element (42.76%, ARE), MYB binding site involved in drought-inducibility (27.59%, MBS), low temperature-responsive (8.28%, LTR) element and stress-responsive element (21.38%, TC-rich repeats) (**Figure 6**; **Table S6**). Except for *AhPLDα8B3* and *AhPLDδ2A*, almost all *AhPLD*s contained at least one stress-responsive element, indicating *AhPLD*s played an important role not only in peanut growth and development, but also in stress responses. The simultaneous appearance of stress and hormone related *cis*-elements in some *AhPLD*s suggesting that *AhPLD*s could mediate the cross-talk of stress and hormone signaling pathways and may have potential role in hormone mediated abiotic stress signaling in peanut.

### Protein-protein interactions and miRNA-genes regulatory networks of peanut PLDs

**Figure 7:**
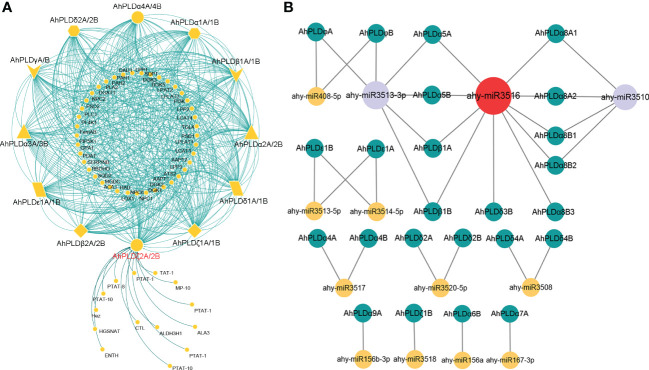
Interactions and regulations of peanut *PLD* genes with other proteins and miRNAs. **(A)** Protein-protein interaction network of peanut PLD proteins with other proteins. The yellow geometric figures represent peanut PLD proteins. The little yellow dots represent other proteins that interact with AhPLDs. The interaction relationships are shown by green lines. **(B)** Regulatory network of putative miRNAs and their targeted *AhPLDs*. The yellow, purple and red circles represented miRNAs. The green circles represent targeted *AhPLDs*. The putative regulatory relationships between miRNAs and their targeted *AhPLDs* are shown in grey lines.

To elucidate the metabolic regulation network mediated by PLDs in peanut and further understand the biological function of *AhPLDs*, the protein-protein interactions (PPIs) of AhPLDs were analyzed using ortholog-based method. Totally, 24 AhPLDs had orthologous relationships with 12 *Arabidopsis* PLDs and interacted with 55 functional proteins (**Figure 7A**; **Table S7**). As expected, most proteins were the functionally validated components of lipid biosynthetic and lipid metabolic processes, such as amino alcohol-phosphotransferase (AAPT), diacylglycerol O-acyltransferase 1 (DGAT1), diacylglycerol kinases (DGKs), lysophosphatidyl acyltransferase 2 (LPAT2) and triglyceride lipase (TGL4). Some others like phospholipase C1 (PLC1), phosphatidylinositol 3-kinase (PI3K), phosphatidylinositol 4-OH kinase 1 (PI4K1) and phosphatidylinositol-4-phosphate 5-kinase 1 (PIP5K1) mainly participated in the phosphatidylinositol-mediated signaling. Besides, several AhPLDs could interact with phospholipid/glycerol acyltransferase family protein ATS2, G protein alpha subunit 1 (GPA1), lipoxygenase 1 (LOX1) and plasma-membrane choline transporter-like protein CTL1. These proteins may regulate plant root development, pollen tube growth, post-embryonic development, seed development and other system development processes.

Moreover, the pathways in response to stress or abiotic stimulus were also significantly enriched by abundant proteins that interacted with AhPLDs, for example, there were five proteins (DGAT1, DGK2, PLC1, respiratory burst oxidase homologue D (RBOHD) and phospholipid-transporting ATPase (PTAT-1)) involved in the response to temperature stimulus, four proteins (DGAT1, PI3K, PLC1 and aldehyde dehydrogenase 3H1 (ALDH3H1)) involved in the response to salt stress, four proteins (DGAT1, PI3K, PLC1 and RBOHD) involved in the response to osmotic stress, three proteins (phosphatidate phosphatase PAH1, monogalactosyldiacylglycerol synthase type C (MGDC) and sulfoquinovosyldiacylglycerol 2 (SQD2)) involved in the response to phosphate starvation, and six proteins (four DGKs, phosphatidic acid phosphatase 1 (LPP1) and RBOHD) involved in the defense response. Notably, there were 14 predicted proteins only interacted with AhPLDζ2A/2B and mainly responsible for lipid transport and phospholipid translocation.

Furthermore, the putative miRNA-targeted *AhPLDs* were predicted to obtain the potential association between lipid metabolic pathways and miRNA regulations. As shown in **Figure 7B**, a total of 24 *AhPLDs* were targeted by 13 miRNAs, of which 12 *AhPLDs* were targeted by only one miRNA, and 12 *AhPLDs* were targeted by two miRNAs. These miRNAs belonged to 12 different families. The comprehensive data of all miRNAs targeted sites/genes was given in **Table S8**. Most miRNAs only targeted one or two *AhPLDs*, but ahy-miR3516, ahy-miR3510 and ahy-miR3513-3p could target ten (*AhPLDα5A/5B*, *AhPLDα8A1/8A2/8B1/8B2/8B3*, *AhPLDβ1A/1B* and *AhPLDδ3B*), six (*AhPLDα5A/5B*, *AhPLDβ1A/1B* and *AhPLDφA/B*) and four *AhPLDs* (*AhPLDα8A1/8A2/8B1/8B2*), respectively, indicating these three miRNAs may be crucial in regulating the lipid metabolic processes.

### Spatial expression profiles of *PLD* genes in peanut

**Figure 8:**
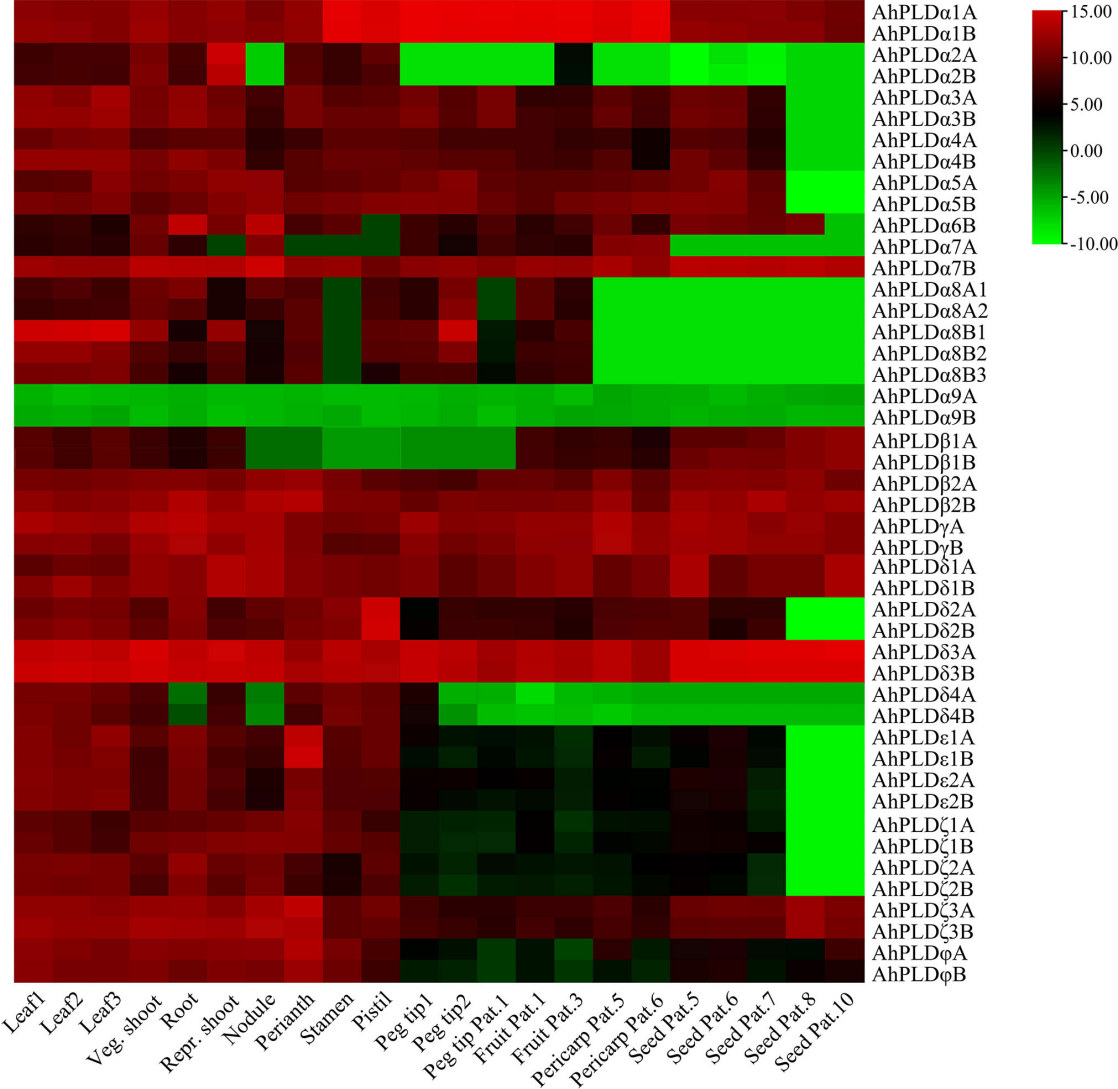
Expression profiles of 46 *AhPLDs* in 22 different tissues. The X axis represents various tissues. The Y axis represents 46 *AhPLDs*. The color scale represents Log2 expression values (FPKM).

The tissue-specific expression profiles of *AhPLDs* were investigated by using the RNA-Seq data from Clevenger et al. (2016). A total of 22 different tissues encompassing almost all peanut tissues and developmental stages were analyzed (**Figure 8**; **Table S9**). The results showed that the expression patterns of 46 *AhPLDs* in various tissues were quite different, though the expression levels of most homologous copies from the A genome and the B genome (such as *AhPLDα1A* and *AhPLDα1B*) were similar because of their extremely high similarity of mRNAs and transcript sizes. The *AhPLDα1A*/*1B*, *AhPLDα7B*, *AhPLDβ2A*/*2B*, *AhPLDγA*/*B, AhPLDδ1A*/*1B*, *AhPLDδ3A*/*3B*, *AhPLDζ3A*/*3B* and *AhPLDφA*/*B* were expressed in all 22 tissues, especially the *AhPLDδ3A*/*3B* exhibited higher expressions in all tissues. Conversely, *AhPLDα9A/9B*, the members of PLDα subgroup, had weak or even undetected expression in any tissues. Besides, almost all *AhPLDs* except for *AhPLDα9A/9B* were highly expressed in all above-ground tissues during the vegetative growth stage, indicating *AhPLDs* might play essential roles in the growth and development of peanut seedlings. The expression levels of *AhPLDε*s, *AhPLDζ*s and *AhPLDφ*s in pods and seeds were significantly lower than in leaves, shoots, roots and flowers, suggesting these *AhPLDs* might mainly involve in peanut seedling growth, root elongation and flowering.

Moreover, some *AhPLDs* displayed tissue-specific or preferential expression patterns, for example, the *AhPLDα1A/1B* showed preferential expressions in flowers and pod development; the *AhPLDα2A*/*2B* had higher expressions in shoot at the reproductive growth stage but hardly expressed in pod or seed developmental processes; the *AhPLDα6B* preferred to express in root and nodule; the *AhPLDα7B* maintained obvious expression levels in all 22 tissues, but its homologue *AhPLDα7A* had almost undetected expression in flowers and nearly mature seeds; the *AhPLDδ2A*/*2B* and *AhPLDε1A*/*1B* showed highest expression levels in pistil and perianth, respectively, indicating their significant roles in the floral organ development in peanut; but only a few *AhPLDs* were highly expressed in seed Pat. 8 and seed Pat. 10.

### Expression profiles of peanut *PLD* genes in response to abiotic stresses

**Figure 9:**
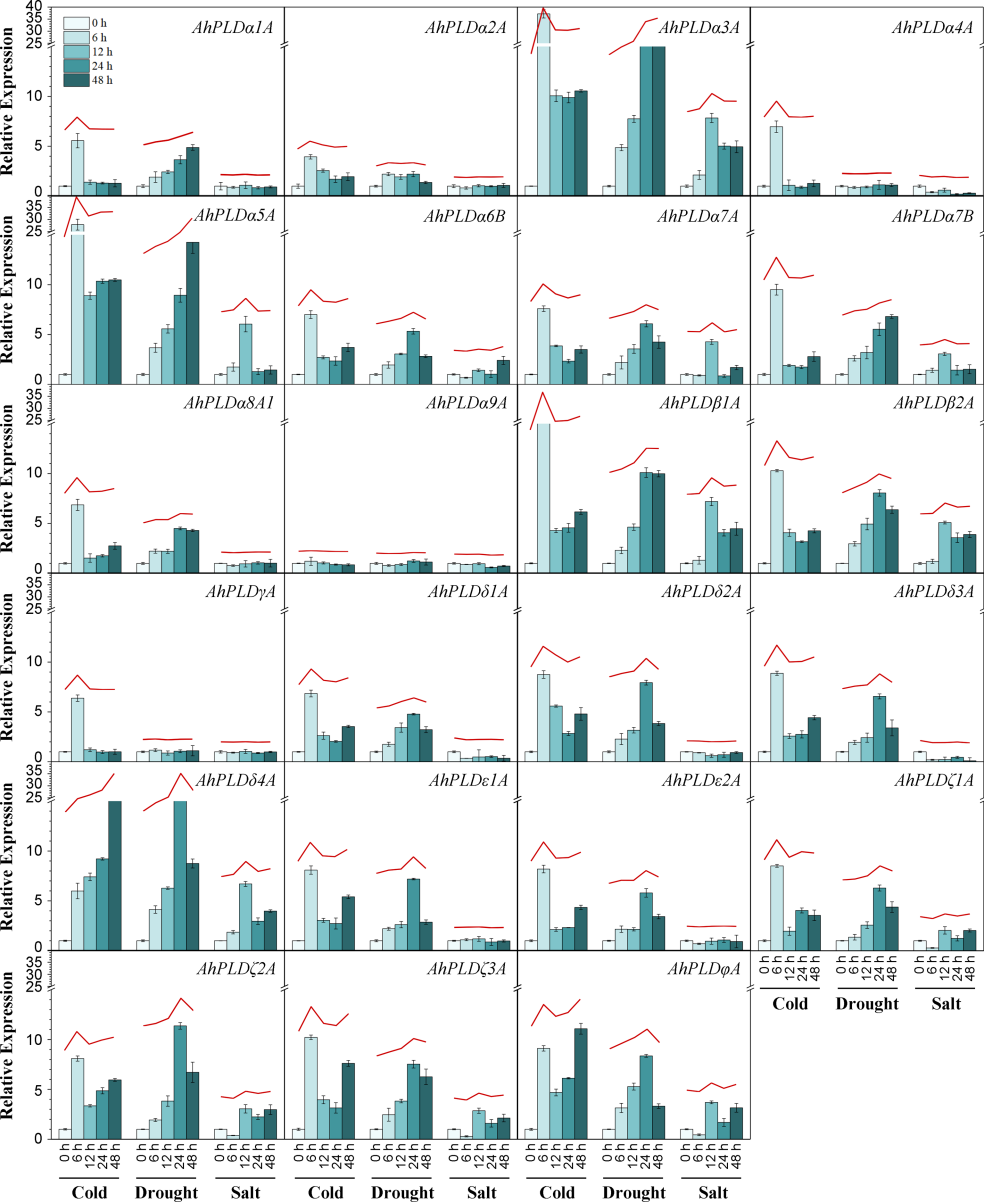
Expression profiles of *AhPLDs* under abiotic stresses. The expression levels of *AhPLDs* under cold, drought and salt were generated using qRT-PCR. The X axis represents different treatments. The Y axis represents relative expression levels of *AhPLDs*. Each bar represents average of three replicates. Standard error is indicated by error bars. The red lines above bars represent the overall trends of *AhPLDs* expressions under different abiotic stresses.

To explore the potential involvement of peanut *PLD* genes in abiotic stresses, their expression profiles under cold, drought and salt were investigated by qRT-PCR (**Figure 9**; **Table S10**). Since the paralogous pairs of *AhPLDs* displayed highly similar nucleotide sequence and expression pattern in 22 different tissues, only *AhPLDs* from the A subgenome were selected for analysis. Similarly, most *AhPLDs* within the same phylogenetic subgroup were also similar in nucleotide sequence and expression pattern, so one representative *AhPLD* in each subclade was chosen for analysis, such as *AhPLDα8A1*. Besides, given that *AhPLDα7B* showed distinct expression pattern in 22 different tissues compared with its homologue *AhPLDα7A*, it was also included. Finally, the expression profiles of 23 *AhPLDs* under three major abiotic stresses were analyzed. The results in **Figure 9** showed that all *AhPLDs* except for *AhPLDα9A* could be induced by at least one abiotic stress, of which 13 *AhPLDs* (*AhPLDα3A*, *AhPLDα5A*-*7A*, *AhPLDβ1A*, *AhPLDβ2A*, *AhPLDδ1A*, *AhPLDδ3A*, *AhPLDδ4A*, *AhPLDζ1A*-*3A* and *AhPLDφA*) were found to be induced by all three abiotic stresses commonly, and seven *AhPLDs* (*AhPLDα1A*, *AhPLDα2A*, *AhPLDα7B*, *AhPLDα8A1*, *AhPLDδ2A*, *AhPLDε1A* and *AhPLDε2A*) could be induced by both cold and drought stresses, and one *AhPLD* gene (*AhPLDα4A*) could be induced by both cold and salt stresses, and *AhPLDγA* was only induced by cold stress. Surprisingly, almost all *AhPLDs* induced by cold and drought were up-regulated compared with controls.

Under cold stress, the expressions of *AhPLDα3A*, *AhPLDα5A*, *AhPLDβ1A*, *AhPLDδ4A*, *AhPLDζ3A* and *AhPLDφA* were most significantly up-regulated. Among them, *AhPLDδ4A* showed a continuous increase with the prolongation of cold time, the fold change (FC) at 48 h of cold stress reached 23.48; *AhPLDα3A*, *AhPLDα5A* and *AhPLDβ1A* showed a trend of increasing (0-6 h) first and then decreasing (6-48 h), peaking at 6 h of cold stress with the FC values of 37.19, 27.86 and 16.11, respectively; similarly, *AhPLDζ3A* and *AhPLDφA* also showed a trend of increasing first and then decreasing, while their expression level was increased again after 48 h of cold stress. Under drought stress, the expressions of most *AhPLDs* were significantly up-regulated in the early stage (24 h) of stress condition, but their expressions did not continue to increase with the duration of stress, typically like *AhPLDδ4A*, *AhPLDζ2A* and *AhPLDφA*; while such *AhPLDs* as *AhPLDα3A* (19.36 FC), *AhPLDα5A* (14.25 FC) and *AhPLDβ1A* (9.99 FC) kept up-regulated expression to 48 h of drought stress. Compared with cold and drought, the transcript levels of *AhPLDs* under salt tress were lower, some of which were even not differentially expressed or down-regulated. For example, *AhPLDα3A* (7.84 FC), *AhPLDα5A* (6.05 FC), *AhPLDβ1A* (7.20 FC), *AhPLDβ2A* (5.09 FC) and *AhPLDδ4A* (6.70 FC) were found to be induced to highly express exclusively at 12 h of salt treatment, the expressions of *AhPLDζ1A*, *AhPLDζ2A*, *AhPLDζ3A* and *AhPLDφA* were down-regulated at 6 h but up-regulated with the duration of salt stress, whereas *AhPLDα4A*, *AhPLDδ1A* and *AhPLDδ3A* were continuously down-regulated with the log_2_FC values of -1.74, -1.47 and -3.32 at 48 h, respectively. Above fundings suggested that *AhPLDs* were potentially involved in signaling triggered by multiple abiotic stresses, and many of them may act as positive regulators of cold and drought stresses in peanut, especially such as *AhPLDα3A*, *AhPLDα5A*, *AhPLDβ1A* and *AhPLDδ4A*.

## Discussion

As one of the most representative phospholipases in plants, PLD can catalyze the hydrolysis of membrane lipids for lipid remodeling and mediate many physiological processes in plant growth, development and responses to abiotic stresses (Wang, 2005). The identification and functional validation of the *PLDs* may hold the promise to breed the improved crops with excellent agronomic traits and stress tolerances to combat the challenge of global climate change. In this study, we obtained 46 *AhPLDs* in allotetraploid peanut as well as 22 *AdPLDs* and 22 *AiPLDs* in its two diploid progenitors *A. duranensis* and *A*. *ipaensis*, respectively (**Table 1**). Obviously, the number of *PLDs* in *A. hypogaea* was greater than already reported plant species (Qin and Wang, 2002; Li et al., 2007; Liu et al., 2010; Zhao et al., 2012; Lu et al., 2019; Sagar et al., 2021), which might be due to the hybridization of *A. duranensis* and *A*. *ipaensis* with succeeding polyploidization. The similar results are also reported in cotton (Tang et al., 2016a; Tang et al., 2016b). Based on the conserved structural domains and sequence properties, these 90 *Arachis PLD* genes could be divided into seven subgroups with distinct biochemical, regulatory and catalytic properties, including α, β, γ, δ, ε, ζ and φ isoforms but excluding isoform κ (**Figure 2**). At present, PLDκ has only been identified in rice and encodes a C2-PLD (Li et al., 2007).

The specific patterns of retention or dispersion of family genes are vital clues to understand the homoeologous chromosome interaction and genetic evolution during plant allo-polyploidization. In this study, the integrated results of phylogeny, gene structure and gene duplication showed that most allotetraploid peanut *PLDs* had at least two homoeologous copies in A and B subgenomes as well as orthologous genes with its progenitors (**Figures 3**–**5**). It proved that a specific large-scale genome duplication event has occurred during the peanut origin, with the segmental duplication and tandem duplication jointly taking place at some locations (**Figure 5**; **Table S4**). But the orthologous gene of *AhPLDα6B* was not found in peanut genome, suggesting a *AhPLDα6A* loss event occurred during peanut evolution. Like *Arabidopsis* and other plant species, the small clade of PLDγs was closer to the clades of PLDβs and PLDδs on the phylogenetic tree (**Figure 2**), and they contained similar numbers of exons and introns (**Figure 3**). This suggested that the isoforms β, γ and δ might have originated as one group in plants but be separated into different functional isoforms during the evolution due to the possible gene duplication events. Similarly, α and ε isoforms might also originate from the common ancestor. But PLDζs and PLDφs had dissimilar intron numbers and belonged to the PX/PH-PLD and SP-PLD subfamilies, respectively, suggesting their convergent evolution *via* two independent evolutionary paths. Besides, the conservation of isoform φ could be also proved by the rate of molecular evolution, in which the *Ka*/*Ks* ratios of gene pairs in subgroup PLDφ were smaller than those in other subgroups (**Table S5**).

Based on the sequence similarity, structural conservation and close evolutionary relationships of ortholog genes among different species, a functional conservation of peanut *PLDs* might also be predicted. The PPI network of AhPLDs showed that most proteins that interacted with AhPLDs (like non-specific phospholipase C (NPC), DGAT1 and DGKs) were the major components of lipid biosynthesis, lipid metabolism and lipid signaling pathways (**Figure 7A**; **Table S7**), which proved the central and conservative functions of peanut PLDs in lipid-related biological processes. Currently, it has become increasingly difficult to find an area of cell biology in which lipids do not have important roles as signaling and regulatory molecules (Hou et al., 2016). For example, *DGAT1*, *DGK2*, *DGK3* and *DGK5* could enhance plant cold tolerance by balancing triacylglycerol (TAG) and PA production (Tan et al., 2018); *NPC4* knockout plants displayed increased sensitivity to salt stress in root elongation, seedling biomass, and seed germination (Kocourkova et al., 2011); PLDζs could hydrolyze phosphatidylcholine to supply phosphorus for cell metabolism and DAG for galactolipid synthesis during phosphorus starvation (Lin et al., 2020). Besides, AhPLDs also interacted with other proteins such as protease inhibitors, GPA1 and RBOHD (**Figure 7A**, **Table S7**). These interactions can regulate PLD activity and intracellular locations, thus affecting cellular functions. For instance, PLDα1 could interact with the GPA1 through its DRY motif to mediate ABA signaling in *Arabidopsis* (Zhao and Wang, 2004). Furthermore, there were 24 *AhPLDs* targeted by 13 miRNAs, suggesting the complex regulation network of *AhPLDs* involved in and providing the clues to genetically engineer *AhPLDs* precisely through miRNA mediation.

Hormones are important regulators of plant growth and development. Many basic biological processes in peanut, such as seed germination, root hair growth, pollen tube elongation, blossom and leaf senescence, are known to be regulated by auxin, ABA, gibberellic acid (GA), JA and ethylene (Wang et al., 2018; Guo et al., 2021). Here, the *AhPLDs* expressed in various peanut tissues were found to contain at least one class of hormone-responsive *cis*-elements in their respective promoters (**Figure 6**; **Table S6**). *AhPLDα1A/1B* that contained ABA-responsive elements could be expressed in all peanut tissues and showed preferential expression in flowers and pod development. In *Arabidopsis*, PLDα1 can interact with a low-affinity nitrate transporter NRT1.2 to positively regulate ABA-mediated seed germination and seedling development (Li et al., 2020). It proved that the regulation of *AhPLDα1A/1B* on peanut growth and development may be mediated by ABA. Isoform PLDδ is the second abundant subfamily next only to isoform PLDα. It represents the majority of PLD isoforms expressed in male gametophyte throughout angiosperms evolution and has been found to be expressed higher in old leaves, stem, roots and flowers than in young leaves and siliques (Qin and Wang, 2002). Here, all *AhPLDδ*s had high expression levels in perianths, stamens and pistil, suggesting their central roles in peanut floral organ development. *AhPLDδ3A/B* even showed high expressions in all 22 tissues. This might be supported by the abundant hormone-responsive elements in their promoters. In tobacco (*Nicotiana tabacum* L.), PLDδ3 is the most important member active in pollen tubes. Tightly controlled production of PA generated by PLDδ3 is crucial for maintaining the balance between various membrane trafficking processes that are vital for plant cell tip growth (Pejchar et al., 2020). However, *AhPLDα9A* did not contain any *cis*-regulatory elements that respond to hormones, which may be the major factor that caused its non-expression in all peanut tissues. There is also evidence that *PLD* genes can be induced by ethylene to regulate the programmed death of plant cells (Lanteri et al., 2008), but no ethylene-responsive element could be found in any peanut PLD promoters here.

Besides, many *cis*-elements in response to diverse environmental stimuli were also found in *AhPLD*s’ promoters, including ARE, MBS, LTR and T-rich (**Figure 6**; **Table S6**). It has been proved that abiotic stresses, such as cold, drought and salinity, could trigger high expressions of most *PLDs* low or weakly expressed under normal growth conditions, as a result of that a number of specific elements are located in their promoters (Wei et al., 2022). As expected, almost all *AhPLD*s (except for *AhPLDα9A*) could be induced by specific or multiple adversities (**Figure 9**), proving their potential roles in abiotic stress tolerances. At present, the functions of 14 *AhPLD*s’ *Arabidopsis* orthologs in stress resistances have been determined (**Table 2**). For example, *AtPLDα1* (the ortholog of *AhPLDα1A*/*B*) has been found to participate in ABA signaling and responses to cold, drought and salt stress (Bargmann et al., 2009); *AtPLDα3* (the ortholog of *AhPLDα3A*/*B*) knockout mutant plants exhibit high sensitivity towards salinity, dehydration and ABA, while gain-of-function of *AtPLDα3* leads to reduced sensitivity in transgenic plants (Hong et al., 2008); *AtPLDδ* (the ortholog of *AhPLDδ1A/1B*) also has been proved to regulate stress resistances, such as freezing, severe dehydration, high salt, oxidative assault and ultra-violet irradiation (Zhang et al., 2003; Li et al., 2004; Liu et al., 2021); *AtPLDδ* and/or *AtPLDα1* can form a regulatory feedback loop with MPK3 and MPK6 to regulate PLD stability and submergence-induced PA production (Zhou et al., 2022).

**Table 2:** Orthologous *PLD* genes in peanut and *Arabidopsis* with known abiotic stress tolerant functions.

AhPLDs	Orthologs	Functions	References
*AhPLDα1A/1B*	*AtPLDα1* (*AT3G15730*)	Salt and ABA responses; drought tolerance; freezing tolerance; hypoxia signaling; high-Mg_2_^+^ stress response; wounding response; mediating superoxide production;	Sang et al., 2001; Bargmann et al., 2009; Hou et al., 2016; Kocourkova et al., 2020; Zhou et al., 2022
*AhPLDα2A/2B*	*AtPLDα2* (*AT1G52570*)	Heat stress memory	Urrea Castellanos et al., 2020
*AhPLDα3A/3B*	*AtPLDα3* (*AT5G25370*)	Hyperosmotic response	Hong et al., 2008
*AhPLDα4A/4B*	*AtPLDα4*/ε (*AT1G55180*)	Hyperosmotic response; nitrogen deficiency response	Hong et al., 2016; Yao et al., 2022
*AhPLDγA/B*	*AtPLDγ1* (*AT4G11850*)	Wounding response	Wang et al., 2000
*AhPLDδ1A/1B*	*AtPLDδ* (*AT4G35790*)	Guard cell signaling and drought tolerance; hypoxia signaling; osmotic stress-triggered stomatal closure; salt stress tolerance; heat stress defense; freezing tolerance	Li et al., 2004; Angelini et al., 2018; Liu et al., 2021; Song et al., 2021; Zhou et al., 2022
*AhPLDζ1A/1B*	*AtPLDζ1* (*AT3G16785*)	Phosphate deficiency response; salt stress response	Li et al., 2006; Korver et al., 2020
	*AtPLDζ2* (*AT3G05630*)	Phosphate deficiency response; root hydrotropism under drought stress; salt stress response	Li et al., 2006; Taniguchi et al., 2010; Su et al., 2018

In this study, there were five *AhPLD*s (*AhPLDα3A*, *AhPLDα5A*, *AhPLDβ1A*, *AhPLDβ2A* and *AhPLDδ4A*) found to be highly up-regulated under all three abiotic stresses commonly, suggesting these five *AhPLD*s might be involved in multiple regulatory pathways at the same time and lead to a wider range of stress resistances in peanut. But the up-regulated expressions of *AhPLDα1A* and several other *AhPLD*s were only induced by cold and drought rather than salinity. *AhPLDα4A*, *AhPLDδ1A* and *AhPLDδ3A* were even continuously down-regulated under salt stress. These results suggested that most *AhPLD*s were mainly involved in cold and drought tolerances but had little or even negative regulation on salt tolerance of peanut. Besides, *AhPLDγA* was significantly up-regulated only at the early stage (6 h) of cold stress, which may be caused by the low-temperature responsive element (LTR) in its promoter. *AhPLDα9A* could neither be expressed in any peanut tissues, nor be induced by any abiotic stresses in peanut. This deviation proved that *AhPLDα9A* might have functional roles in some processes other than peanut growth or stress signaling and its specific biological mechanism need to be further studied.

## Conclusion

In conclusion, a total of 22, 22 and 46 *PLD* genes were identified in *A. duranensis*, *A. ipaensis* and *A. hypogaea*, respectively. Our comparative analyses provided valuable insight into the understanding of sequence characteristics, conserved domains, phylogenetic relationships and molecular evolution of PLD genes in allotetraploid peanut and its diploid progenitors. The predictive analytics of *cis*-regulatory elements, protein-protein interactions, putative miRNA expanded the view of transcriptional regulation and potential functions of *AhPLD* genes. Importantly, the expression patterns of tissue-specific and abiotic stress-responsive *AhPLD*s obtained from RNA-seq and qRT-PCR results offered useful information for further functional investigations. Several candidate *AhPLD*s, such as *AhPLDα3A*, *AhPLDα5A*, *AhPLDβ1A*, *AhPLDβ2A* and *AhPLDδ4A*, can be utilized for genetic manipulation of peanut and other legume crops for improved abiotic stress tolerance and productivity.

## Data availability statement

The datasets presented in this study can be found in online repositories. The names of the repository/repositories and accession number(s) can be found in the article/**Supplementary Material**.

## Author contributions

HZ and HY designed the research study. YY, SW and CZ conducted the bioinformatics analysis. JY, XA, NZ and XL performed the experiments. HZ and XZ analyzed the data. HZ and HY wrote and revised the manuscript. All authors contributed to the article and approved the submitted version.
