# Supplementary figures and images for: Genome-wide characterization of phospholipase D family genes in allotetraploid peanut and its diploid progenitors revealed their crucial roles in growth and abiotic stress responses
Source: Front Plant Sci. 2023 Jan 20;14:1102200. doi: 10.3389/fpls.2023.1102200 (PMC9895952; doi:10.3389/fpls.2023.1102200)

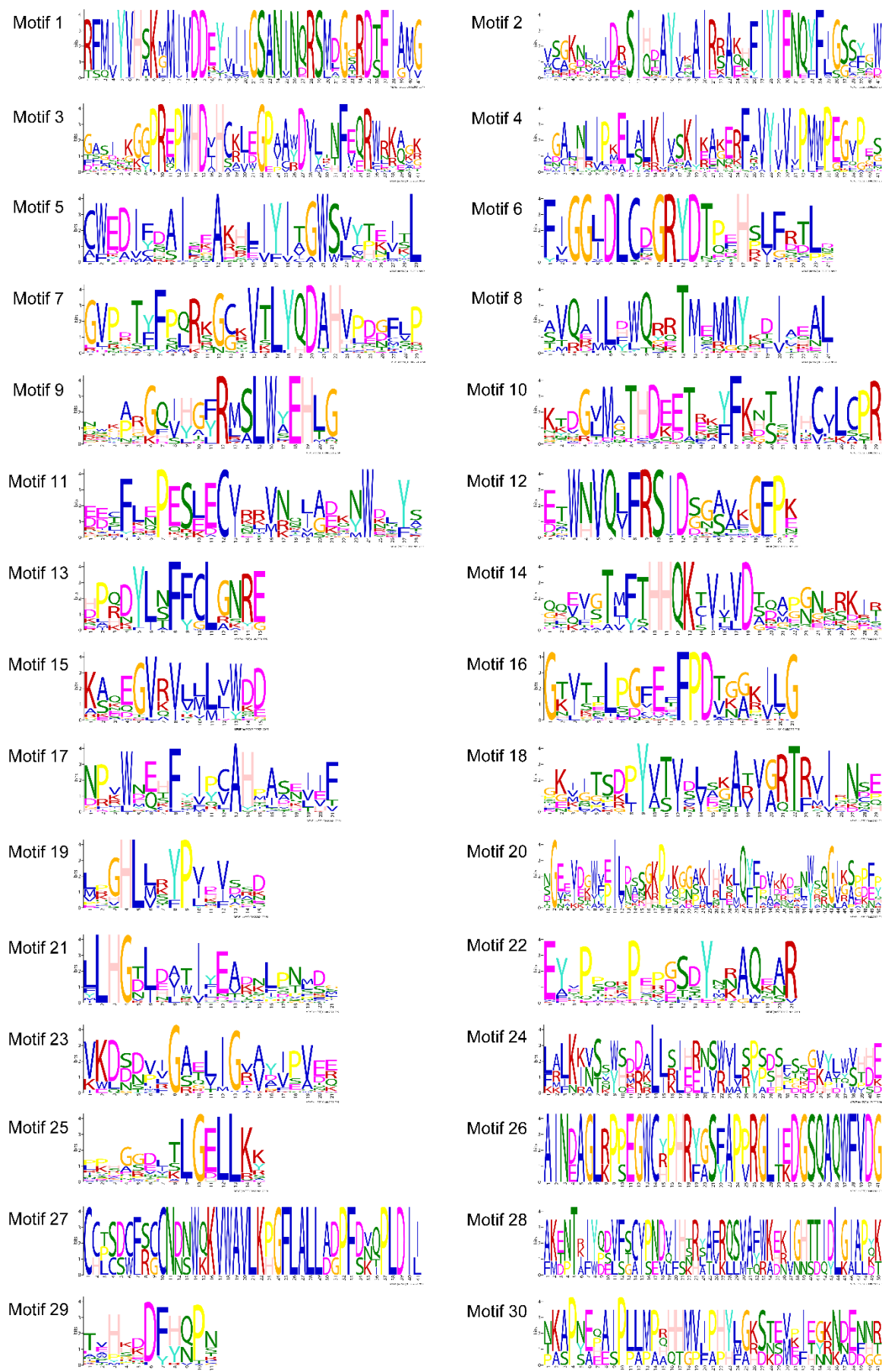

**Figure S1** Sequence logos for the 30 motifs of *Arachis* PLD proteins

Supplement: Supplementary file 1 [file DataSheet_1.pdf]
